# Association between circulating miRNAs and spinal involvement in patients with axial spondyloarthritis

**DOI:** 10.1371/journal.pone.0185323

**Published:** 2017-09-22

**Authors:** Klára Prajzlerová, Kristýna Grobelná, Markéta Hušáková, Šárka Forejtová, Astrid Jüngel, Steffen Gay, Jiří Vencovský, Karel Pavelka, Ladislav Šenolt, Mária Filková

**Affiliations:** 1 Institute of Rheumatology and Department of Rheumatology, 1st Faculty of Medicine, Charles University, Prague, Czech Republic; 2 Center of Experimental Rheumatology, University Hospital Zurich, Zurich, Switzerland; Kunming University of Science and Technology, CHINA

## Abstract

**Objectives:**

Dysregulation of miRNAs and their target genes contributes to the pathophysiology of autoimmune diseases. Circulating miRNAs may serve as diagnostic/prognostic biomarkers. We aimed to investigate the association between circulating miRNAs, disease activity and spinal involvement in patients with axial spondyloarthritis (AxSpA).

**Methods:**

Total RNA was isolated from the plasma of patients with non-radiographic (nr)AxSpA, patients with ankylosing spondylitis (AS) and healthy controls (HC) via phenol-chloroform extraction. A total of 760 miRNAs were analysed with TaqMan^®^ Low Density Arrays, and the expression of 21 miRNAs was assessed using single assays.

**Results:**

Comprehensive analysis demonstrated the differential expression of miRNAs among patients with progressive spinal disease. Of the 21 miRNAs selected according to their expression patterns, the levels of miR-625-3p were significantly different between nr-AxSpA patients and HCs. We found no correlation between miRNA levels and Bath Ankylosing Spondylitis Disease Activity Index (BASDAI) in nr-AxSpA patients. Selected miRNAs, such as miR-29a-3p, miR-146a-5p or miR-222-3p with an established role in extracellular matrix formation and inflammation were associated with spinal changes and/or disease activity assessed by BASDAI in AS patients, including miR-625-3p reflecting disease activity in AS with spinal involvement.

**Conclusions:**

Our data indicate that circulating miRNAs play a role in the pathogenesis of AxSpA and are also suggestive of their potential as biomarkers of disease progression. We hypothesize that differential systemic levels of miRNA expression reflect miRNA dysregulation at sites of spinal inflammation or bone formation where these molecules contribute to the development of pathophysiological features typical of AxSpA.

## Introduction

Axial spondyloarthritis (AxSpA) is a chronic inflammatory disease that mainly affects the axial skeleton, sacroiliac joints and entheseal spinal structures. It encompasses patients with ankylosing spondylitis (AS) with radiographic sacroiliitis and syndesmophytes, as well as patients with early or abortive forms of spondyloarthritis (SpA) characterized by the presence of sacroiliac inflammation detected by magnetic resonance imaging (MRI) or the presence of HLA-B27 in combination with features characteristic of SpA [1, 2]. AS is an inflammatory disease characterized by new bone formation. Mononuclear cells and osteoclasts initiate local osteitis, which leads to cartilage erosion and bone destruction, as well as osteoblast differentiation and subsequent syndesmophyte formation [3, 4].

Inflammation develops several years before structural damage becomes visible on plain radiographs. Although patients may have longstanding symptoms, the diagnosis of AS based on the modified New York criteria delays early treatment, as radiographic sacroiliitis represents a late sign of disease. Therefore, the Assessment of SpondyloArthritis International Society (ASAS) developed new classification criteria for the diagnosis of AxSpA that takes non-radiographic (nr-AxSpA) findings into account [5]. Shorter disease duration, younger age, elevated baseline C-reactive protein (CRP) levels and active inflammatory changes involving the sacroiliac joint are associated with better responses to anti-TNF therapy in patients with nr-AxSpA [6]. Therefore, early diagnosis, disease monitoring and therapeutic response prediction are very important.

Several biomarkers have been tested regarding their usefulness in diagnosing disease, monitoring disease activity and predicting therapeutic responsiveness but have thus far not been implemented in clinical practice [7]. HLA-B27 remains the best genetic biomarker for diagnosing AxSpA, and CRP remains the best circulating marker for assessing disease activity and predicting treatment responsiveness and structural progression [7].

MicroRNAs (miRNAs) are small, non-coding RNAs that function as post-transcriptional regulators of gene expression. Altered miRNA expression and target gene dysregulation have been shown to contribute to the pathophysiology of many autoimmune diseases, including rheumatic diseases [8]. Although the (patho) physiological roles of circulating miRNAs remain largely unknown, cell-free circulating miRNAs appear to be promising disease biomarkers [9]. While rheumatoid arthritis (RA) has been extensively investigated, comprehensive studies regarding miRNAs in patients with AxSpA are lacking.

The aim of the present study was to identify circulating miRNAs in patients with AxSpA and to investigate their association with disease characteristics, including spinal disease severity.

## Material and methods

### Patients

This study included 20 patients with nr-AxSpA, 24 AS patients with isolated sacroiliitis without spinal involvement (AS stage I), 24 patients with AS with spinal involvement (presence of syndesmophytes, AS stages II-V), including 7 patients with a bamboo spine, and 29 healthy controls (HC). Radiographic staging was performed as previously described [10]. All patients fulfilled the 2011 ASAS classification criteria for the diagnosis of AxSpA [11]. Disease activity was assessed using the Bath Ankylosing Spondylitis Disease Activity Index (BASDAI) [12] and CRP. The clinical characteristics of the patients and HCs are shown in Table 1. Patients were recruited from the outpatient clinic of the Institute of Rheumatology, Prague in 2013–2014. Written informed consent was obtained from all participants prior to enrolment, and the study was approved by the local Ethics committee at the Institute of Rheumatology in Prague.

**Table 1:** Clinical characteristics of healthy controls and patients with axial spondyloarthritis.

Variable	HC	nr-AxSpA	sacroiliitis	AS II-V
n	29	20	24	24
Gender, female//male, n	9/20	9/11	4/20	4/20
Age, years	34.0 ± 9.2 (20.7, 56.8)	34.9 ± 10.3 (21,3, 68.0)	32.6 ± 8.2 (22.1, 57.9)	40.0 ± 8.3 (25.2, 45.3)
HLA-B27 positivity, n	NA	20	22	21
Disease duration, years	NA	1.6 ± 2.5 (0, 9)	4.3 ± 3.7 (0,14)	6.8 ± 4.3 (0,17)
CRP, mg/l	NA	6.8 ± 7.5 (0.3, 29.0)	18.7 ± 26.3 (1.7, 117.0)	15.0 ± 16.3 (0.5, 77.5)
BASDAI score	NA	3.2 ± 2.3 (0.2, 6.7)	6.0 ± 2.6 (0.1, 9.4)	4.6 ± 2.5 (0.7, 8.2)
Peripheral arthritis, n	NA	4	3	1
Enthesitis, n	NA	9	2	8
Uveitis, n	NA	10	6	9
Treatment, n: NSAID DMARDs Biological therapy	0 0 0	15 4 1	17 5 2	10 2 12

Abbreviations: AS, ankylosing spondylitis; AS II-V, ankylosing spondylitis with spinal involvement; BASDAI, Bath Ankylosing Spondylitis Disease Activity Index; CRP, C-reactive protein; DMARDs, disease modifying antirheumatic drugs; HC, healthy controls; NSAID, non-steroidal anti-inflammatory drug; nr-AxSpA, non-radiographic axial spondyloarthritis; NA, not applicable;. Data are expressed as the mean±SD and minimum and maximum values (min, max).

### Samples and RNA isolation

Whole blood samples collected to EDTA tubes were obtained from all participants and plasma was separated by centrifugation within 4 hours of collection ensuring constant pre-analytical condition for all samples. All plasma samples were stored at -80°C and experienced no freeze-thaw cycles before use. Total RNA was extracted from plasma samples using phenol-chloroform extraction, as previously described [9]. Briefly, 500 μl of plasma was homogenized with 500 μl of Trizol LS reagent (Thermo Fisher Scientific, Waltham, MA, USA) and then centrifuged at 12,000 × g for 10 minutes at 4°C. Three cycles of acid phenol-chloroform (Thermo Fisher Scientific) extraction were performed. RNA was precipitated by adding 5μg of RNase-free glycogen (Roche Diagnostics, Mannheim, Germany) and 100% isopropanol and then incubated for 10 minutes at room temperature before being centrifuged at 12,000 × g for 10 minutes at 4°C. The pellet was washed with 75% ethanol, and RNA was dissolved in RNase-free water. Three synthesized *C*. *elegans* miRNAs, cel-miR-39, cel-miR-54 and cel-miR-238 (Integrated DNA Technologies, Coralville, IA, USA), 25 fmol each, were spiked into plasma samples after denaturation and served as internal calibrators, as previously described [13]. RNA sample quality control was initially performed using Agilent 2100 Bioanalyser with Agilent Small RNA kit (Agilent, CA, USA) as RNA isolation quality measure and then using a NanoDrop 2000c spectrophotometer (Thermo Fisher Scientific) in remaining samples.

### miRNA analysis

First, twenty non-pooled individual samples (5 samples from each group) were analysed using a TaqMan^®^ Low Density Array (Thermo Fisher Scientific). Complementary DNA was obtained by reverse transcription using a TaqMan^®^ MicroRNA Reverse Transcription Kit with Megaplex RT Primers with equal RNA input. cDNA was preamplified using 2x TaqMan^®^ PreAmp Master Mix and Megaplex^™^ PreAmp Primers (all Thermo Fisher Scientific) on a PCR thermocycler (Bio-Rad Laboratories, CA, USA). The expression of 760 miRNAs was measured using Human Pool A+B TaqMan^®^ Low Density Array platforms for microRNAs on a 7900RT-PCR thermocycler (Thermo Fisher Scientific). All steps were performed according to the manufacturer’s instructions. Data were analysed with RQ Manager Software (Life Technologies). The dCt method was used for relative quantification as follows: dCt = Ct(array average)-Ct(miRNA of interest), followed by x-fold change calculations.

All miRNAs exhibiting a minimum 1.5 mean fold difference in expression between at least 2 groups according to across-group comparisons (HC vs. nr-AxSpA vs. AS) were taken forward for pathway analysis and literature search as explained below. In total, 21 miRNAs were selected for further validation using single assays. Total RNA from the remaining non-pooled samples was reverse-transcribed using TaqMan Real Time miRNA specific primers (including primers for cel-miR-39, cel-miR-54 and cel-miR-238) and then amplified by real-time PCR with TaqMan probes and TaqMan Universal PCR Master Mix on a 7900RT-PCR thermocycler (Thermo Fisher Scientific). Data were analysed with RQ Manager Software (Thermo Fisher Scientific). The dCt method was used for relative quantification as follows: dCt = Ct(spike-in average)-Ct(miRNA of interest); therefore, higher dCt values represent higher expression levels of particular miRNAs.

### Statistical analysis

Data are expressed as the mean±SD. One-way ANOVA with post-hoc comparison for multiple comparisons or unpaired T test (with Welch’s correction in case of homogeneity assumption violations) for comparisons between 2 groups were used where applicable. Pearson’s correlation coefficient was used to correlate any two variables. P values less than 0.05 were considered statistically significant. All analyses and graphs were performed and generated, respectively, using GraphPad Prism 5.02 (GraphPad Software, La Jolla, CA).

### Literature search

First, DIANA-mirPath tool was used to analyze clustering of miRNAs and pathways. In the next step, an online search (PubMed, performed in March 2016) of the functions of miRNAs was performed and 21 miRNAs with a hypothesized role in the pathogenesis of AxSpA were selected.

## Results

### Comprehensive analysis of circulating miRNAs

A comprehensive screening of 760 miRNAs was performed using TaqMan Low Density Arrays, as described above. Only miRNAs expressed in all 5 samples were taken forward for the analysis. Overall, 162 miRNAs were detected in HCs, 154 miRNAs were detected in patients with nr-AxSpA, 110 miRNAs were detected in patients with sacroiliitis, and 110 miRNAs were detected in AS patients with spinal involvement (AS II-V). Of those miRNAs, 92 were detected in all tested samples, 10 were detected in HCs, nr-AxSpA patients and AS patients with sacroiliitis and 25 were shared by HC and nr-AxSpA patients (S1 Fig). We found no miRNA unique to AxSpA.

Using the approach described in the Methods, miRNAs exhibiting a minimum 1.5 mean fold difference in expression between at least 2 groups according to across-group comparisons (HC vs. nr-AxSpA vs. AS with sacroiliitis and with spinal involvement) were considered for further analysis (S1 Table).

**Fig 1:**
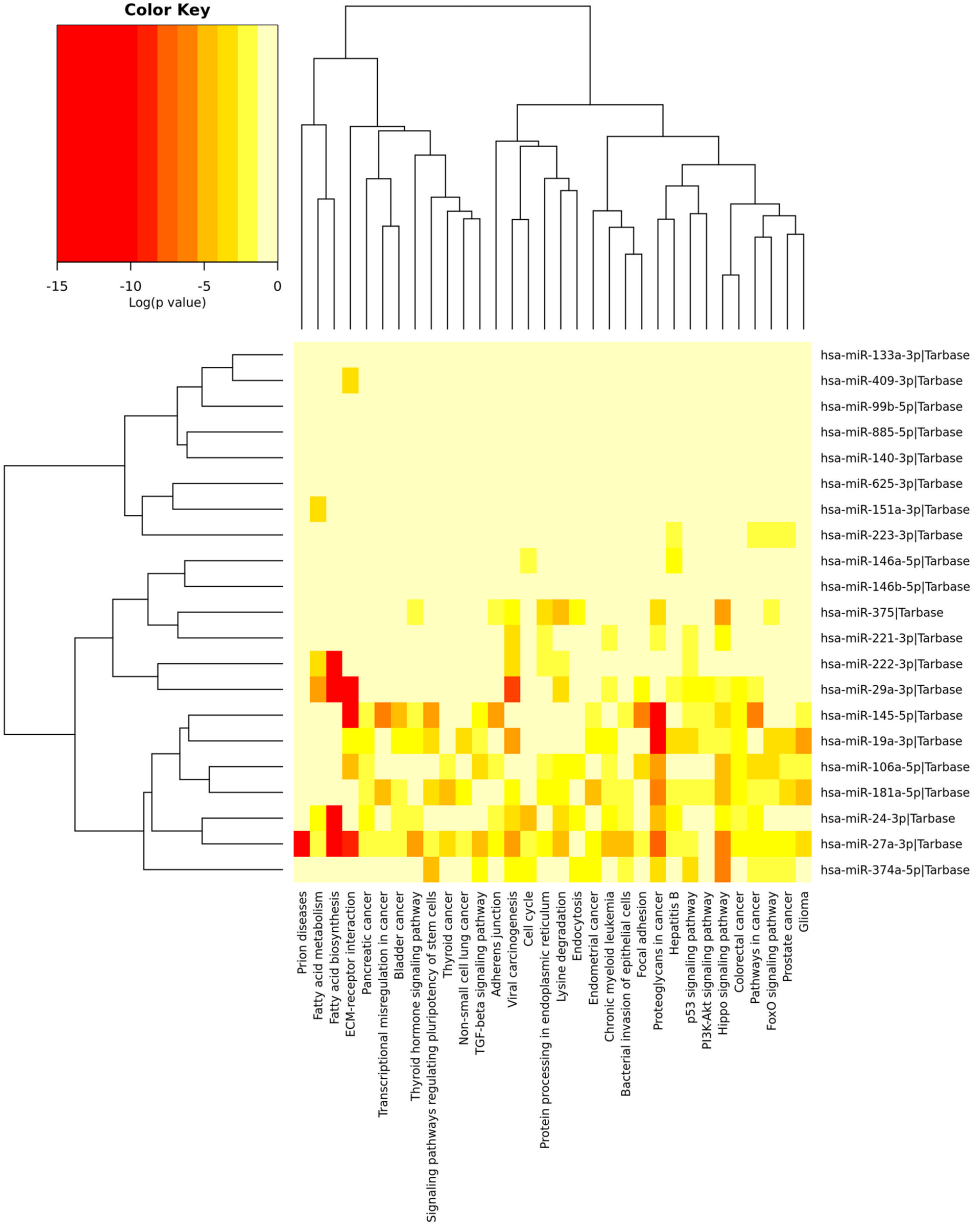
Significance cluster analysis of selected miRNAs using DIANA mirPath tool showing the involvement of miRNAs in different signalling and pathogenic pathways. Although the involvement in certain pathways of several miRNAs overlapped, the function of few miRNAs was unknown and required manual search for their function.

DIANA mir-Path cluster analysis and literature review enabled selection of 21 miRNAs for further validation (Fig 1, S2 Table).

### Differential expression of circulating miRNAs between HC and patients with AxSpA

As mentioned above, 21 selected miRNAs were analysed using single assays to confirm their differential expression (S3 Table).

The expression was compared between HC and patients with AxSpA as follows:

Significantly lower expression (from 1.6 to 3.9 times) of 14 miRNAs most of which are involved in osteoblast differentiation or the Wnt signalling pathway, were noted in all patients with AxSpA irrespective of patient radiographic findings compared to HC (Table 2, extended results shown in S4 Table).

**Table 2:** Summary of expression and function of selected miRNAs as markers of disease activity and hypothesized role in AxSpA.

miRNA	Diagnosis				Disease activity				Treatment response		Hypothesized role in AxSpA
	HC vs. AxSpA^T^	HC vs. nr-AxSpA	HC vs. AS	nr-AxSpA vs. AS	nr-AxSpA	AS	sacroiliitis	AS II-V	NSAID vs. antiTNF	DMARDs. vs. antiTNF	
miR-19a-3p	*	-	**	*	-	-	-	-	-	**	bone formation
miR-24-3p	*	-	**	**	-	-	-	-	***	***	bone formation
miR-27a-3p	**	-	***	**	-	-	-	-	***	**	bone formation
miR-29a-3p	**	-	***	*	-	-	-	BASDAI, CRP	*	**	bone formation,
miR-99b-5p	**	-	***	*	CRP	BASDAI	-	-	***	**	bone formation
miR-106a-5p	-	-	**	**	-	-	-	-	***	***	bone formation
miR-133a-3p	*	-	*	-	-	BASDAI	-	CRP	-	*	differentiation?
miR-140-3p	-	-	*	**	CRP	-	-	-	**	**	inflammation
miR-145-5p	-	-	*	-	CRP	-	-	-	**	*	bone formation
miR-146a-5p	*	-	**	*	-	CRP	-	-	*	*	inflammation
miR-146b-5p	*	-	**	**	-	-	-	-	**	***	migration, invasion
miR-151a-3p	-	-	*	*	-	CRP	-	CRP	-	-	migration
miR-181a-5p	-	-	-	-	-	CRP	-	-	-	-	?
miR-221-3p	-	-	**	*	-	CRP	-	-	-	*	immunopathogenesis
miR-222-3p	***	-	***	*	-	-	-	BASDAI	-	-	bone formation
miR-223-3p	*	-	**	*	-	-	-	-	**	**	bone formation
miR-374a-5p	**	-	***	*	CRP	-	-	-	*	*	bone formation
miR-375	*	-	-	-	-	-	-	BASDAI	-	-	bone formation
miR-409-3p	**	-	***	-	-	-	-	CRP	-	-	proliferation, invasion
miR-625-3p	***	*	***	-	-	BASDAI	-	BASDAI	-	-	?
miR-885-5p	-	-	-	-	-	BASDAI	-	BASDAI	-	-	?

Abbreviations: HC, healthy controls; nr-AxSpA, non-radiographic axial spondyloarthritis; AS, ankylosing spondylitis; AS II-V, ankylosing spondylitis with spinal involvement; DMARDs, disease modifying antirheumatic drugs; NSAID, non-steroidal anti-inflammatory drugs; T, T test; -, not significant;

*p<0.05,

**p<0.01,

***p<0.001.

Statistical significance was calculated using ANOVA unless stated otherwise.

**Fig 2:**
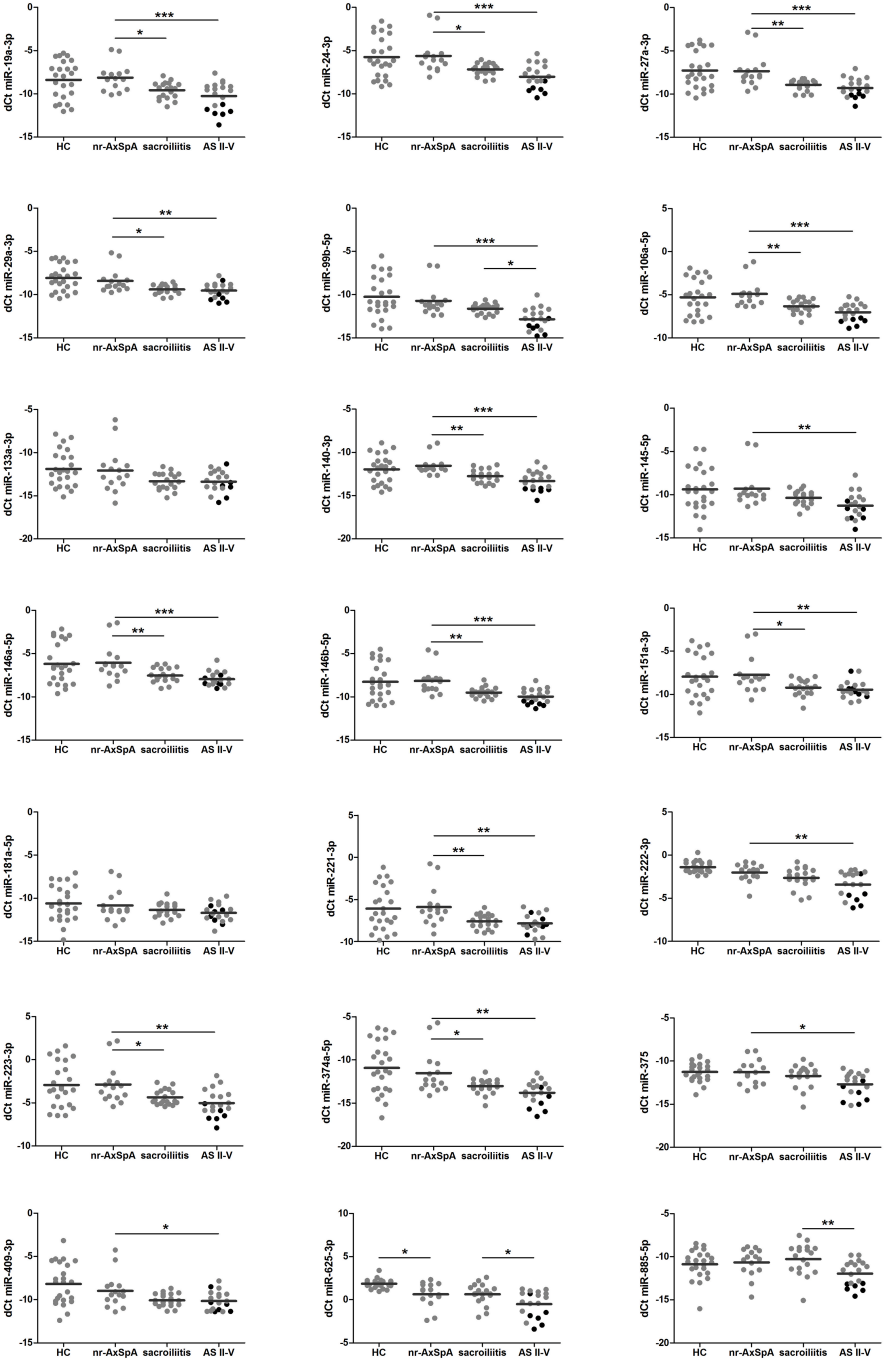
Differences in the levels of circulating miRNAs between healthy controls, patients with non-radiographic AxSpA (nr-AxSpA) and patients with ankylosing spondylitis (AS) with isolated sacroiliitis and spinal involvement (AS II-V). Black symbols indicate patients with bamboo spine. *p<0.05, **p<0.01, ***p<0.001.

As the group of AxSpA is heterogeneous, we next compared patients with AxSpA according radiographic damage with HC (Table 2, S4 Table, Fig 2):

In patients with nr-AxSpA, only miR-625-3p appeared significantly different and exhibited 2.3 times lower expression levels than in HC. Eighteen miRNAs exhibited 2.1 to 5.6 times lower expression levels in radiographic disease irrespective of spinal involvement than in HC, and 14 miRNAs were 2.0–3.9 times lower in AS patients than in patients with non-radiographic disease (Table 2, S4 Table).

These results indicate that some differences exist in the levels of circulating miRNAs between HC and patients with non-radiographic disease, while more differences exist at radiographic stage reflecting bony changes in patients with more advanced disease.

### Effects of spinal involvement on circulating miRNA expression

Next, we evaluated the differences in circulating miRNA levels between patients with nr-AxSpA and definite radiographic disease in patients with isolated sacroiliitis and with spinal involvement (classified as AS II-V) as follows (Fig 2):

**nr-AxSpA vs. AS**: The vast majority of miRNAs (miR-19a-3p, miR-24-3p, miR-27a-3p, miR-29a-3p, miR-106a-5p, miR-140-3p, miR-146a-5p, miR-146b-5p, miR-151a-3p, miR-221-3p, miR-223-3p, miR-374a-5p) exhibited 2.0–5.2 times lower expression levels in both AS groups than in the nr-AxSpA patients. As most of them are associated with bone remodelling, these data indicate that an inverse association exists between radiographic bone formation and circulating miRNA levels.

**Sacroiliitis vs. AS II-V**: However, there were no significant differences in the levels of abovementioned 12 miRNAs between sacroiliitis and AS II-V groups. MiR-99b-5p, miR-625-3p and miR-885-5p exhibited significantly lower expression (2.3 times, 2.2 times, 3.3 times, respectively) in AS patients with spinal involvement (AS II-V) than in those with sacroiliitis. Interestingly, we found no data regarding the roles of miR-625-3p and miR-885-5p in bone formation or inflammation (S2 Table).

Moreover, patients with a bamboo spine exhibited 1.6–8.0 times significantly lower expression levels of some of the miRNAs mentioned above (miR-19a-3p, miR-24-3p, miR-27a-3p, miR-99b-5p, miR-106a-5p, miR-140-3p, miR-145-5p, miR-146a-5p, miR-146b-5p, miR-222-3p, miR-223-3p, miR-374a-5p, miR-375), including miR-625-3p and miR-885-5p than AS patients with less severe spinal damage (Fig 2).

### Association between circulating miRNAs and disease activity

**Fig 3:**
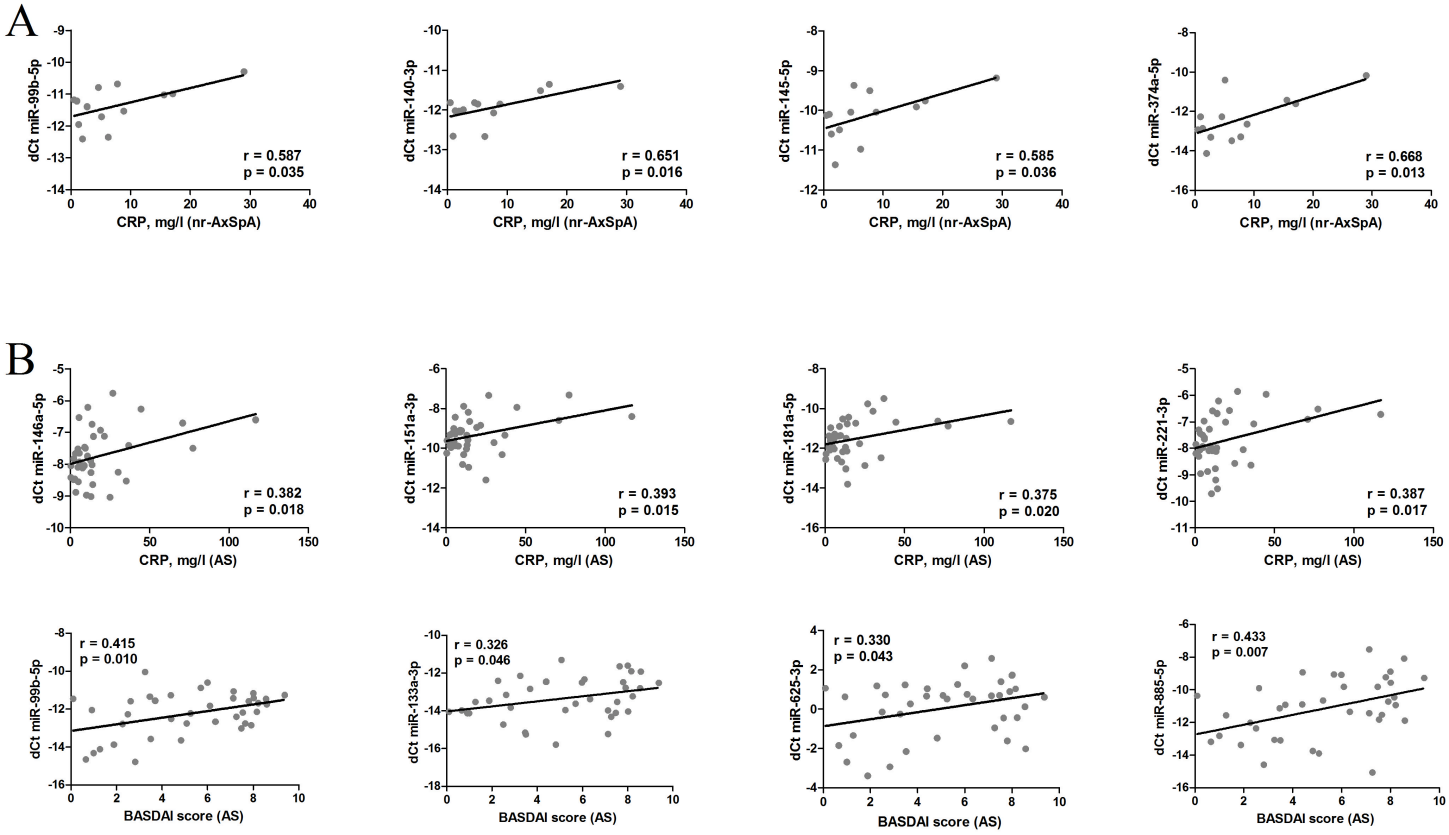
Correlation between circulating miRNAs, CRP and BASDAI in nr-AxSpA (A) and AS patients (B).

We next aimed to analyse the associations between circulating miRNA levels and disease activity parameters (Fig 3).

In patients with nr-AxSpA, no significant associations between miRNA levels and BASDAI were noted. CRP was positively correlated with the levels of miR-99b-5p, miR-140-3p, miR-145-5p and miR-374a-5p expression (Fig 3A).

In all AS patients, BASDAI positively correlated with the levels of miR-99b-5p, miR-133a-3p, miR-625-3p and miR-885-5p, while CRP was correlated with the levels of miR-146a-5p, miR-151a-3p, miR-181a-5p and miR-221-3p (Fig 3B).

When further sub-analysis was performed, we noted no correlations between miRNAs and CRP or BASDAI in patients with sacroiliitis. However, in AS patients with spinal involvement (AS II-V), we noted a positive correlation between BASDAI and mi-29a-3p, miR-222-3p, miR-375, miR-625-3p and miR-885-5p levels and a positive correlation between CRP and miR-29a-3p, miR-133a-3p, miR-151a-3p and miR-409-3p levels.

We then considered different HLA-B27 status, peripheral arthritis or extraarticular manifestations on levels of miRNAs but no effect was found in any of these confounders. While no significant differences were noted in miRNA levels between patients receiving non-steroidal anti-inflammatory drugs (NSAID) or disease modifying antirheumatic drugs (DMARDs), the patients receiving anti-TNF therapy exhibited significantly lower levels of all remaining miRNAs than anti-TNF naïve patients (Table 2, S4 Table).

## Discussion

To our knowledge, this is the first study to perform comprehensive analyses of 760 circulating miRNAs in patients with various stages of AxSpA. We observed differential expression of some miRNAs in patients with more advanced spinal disease.

Circulating miRNAs have been shown to be unexpectedly stable, which makes them accessible via body fluid sampling and potentially useful as biomarkers [13]. Some associations between circulating miRNA levels and disease activity e.g., in early RA [9] or systemic lupus erythematosus [14], have previously been shown; however, data on circulating miRNAs in AxSpA are lacking.

Of 760 screened miRNAs, 21 exhibiting differential expression among patients at various disease stages were selected. We confirmed that 14 miRNAs (miR-19a-3p, miR-24-3p, miR-27a-3p, miR-29a-3p, miR-99b-5p, miR-133a-3p, miR-146a-5p, miR-146b-5p, miR-222-3p, miR-223-3p, miR-374a-5p, miR-375, miR-409-3p and miR-625-3p) had lower expression levels in all AxSpA patients, irrespective of radiographic findings, than in HC. Most of them were shown to be associated with osteoblast differentiation or the Wnt signalling pathway, while others participate in cell differentiation and proliferation or inflammation. These data support our hypothesis that dysregulation of circulating miRNA occurs in patients with AxSpA.

Interestingly, only miR-625-3p was significantly different in nr-AxSpA patients compared to HC. Eighteen miRNAs were confirmed to have lower expression levels in AS patients than in HCs. Of these, 14 exhibited lower expression levels in AS patients with radiographic disease than in AS patients with non-radiographic disease. The levels of 9 miRNAs (miR-19a-3p, miR-24-3p, miR-27a-3p, miR-106a-5p, miR-140-3p, miR-146a-5p, miR-146b-5p, miR-223-3p, miR-374a-5p) were lower in patients with a bamboo spine than in other patients with AS. Interestingly, these 9 miRNAs that exhibited differential expression in conjunction with progressive spinal damage, 6 miRNAs (miR-19a-3p, miR-24-3p, miR-27a-3p, miR-106a-5p, miR-223-3p, miR-374a-5p) play roles in bone formation by mediating Wnt signalling pathway activity, and 3 (miR-140-3p, miR-146a-5p, miR-146b-5p) play roles in inflammation or cell migration that may be related to the pathogenesis of AxSpA.

At present, it is not technically feasible to show the effect of native biofluid circulating miRNA on target genes in tissues using functional experiments due to lack of data on their specific source, trafficking and targeting mechanisms. Drawing firm conclusions on the role of circulating miRNAs in the pathogenesis is therefore difficult and these are mostly inferred from data published on intracellular miRNAs reviewed by literature and databases search. Similarly, we inferred the potential of differentially expressed circulating miRNAs in the pathogenesis of AxSpA based on published data on other diseases.

Many miRNAs shown here to be differentially expressed are involved in bone turnover. MiR-19a was described as a negative regulator of the Wnt signalling pathway in endothelial cells [15]. MiR-24 overexpression significantly inhibited osteogenic differentiation in osteoblastic cells [16]. Runx2, a transcription factor essential for osteoblastogenesis, was shown to negatively regulate the expression of miR-27a [17]. Another study showed that miR-27 promote osteoblast differentiation by modulating Wnt signalling [18]. MiR-106a inhibits osteogenesis in mesenchymal stem cells [19] and is a negative regulator of IL-8, the levels of which are known to be elevated in AS patients [20, 21]. MiR-223 affects bone metabolism, especially osteoclast and osteoblast differentiation [22] and blocking miR-223 inhibits osteoclastogenesis [23]. Moreover, miR-223 appears to be a biomarker of disease activity and treatment response in RA [9, 24]. MiR-374a has been shown to be an activator of the Wnt signalling pathway [25]. In summary, we believe that lower systemic levels of miRNAs may reflect their low local expression levels. Our data show a trend towards lower levels according the extent of spine involvement with the lowest levels in patients with most advanced disease. We hypothesize that lower systemic levels of miRNAs negatively correlate with new bone formation promoted by induction of osteoblastogenesis and to lesser extend also by local inflammation or osteitis initiated by osteoclast infiltration. However, the data regarding miR-27a and miR-374a remain controversial and do not entirely support our theory.

In addition, several miRNAs modulate inflammatory process. IL-1β suppressed miR-140 expression and induced ADAMTS5, a member of the extracellular protease enzyme family. Conversely, transfection of chondrocytes with miR-140 downregulated IL-1β-induced ADAMTS5 expression [26]. In keeping with these findings, miR-140-3p was shown to ameliorate autoimmune arthritis [27]. Administration of miR-146a prevents joint destruction in arthritic mice presenting miR-146a as a negative regulator of inflammation [28]. These data suggest that miR-140-3p and miR-146a dysregulation may be associated with the proinflammatory state characteristic of AxSpA. Also, miR-146a polymorphisms have been suggested to be a potential pathogenic factor for AS [29].

Here we propose circulating miRNAs as markers of disease activity. In nr-AxSpA patients, miR-140-3p, miR-223-3p, miR-99b-5p and miR-145-5p levels correlated with CRP. Both miR-99b and miR-145 were shown to be associated with osteoclast/osteoblast differentiation [30, 31]. However, no associations with BASDAI were observed in these patients or those with sacroiliitis.

Furthermore, in AS patients with spinal involvement, we observed correlations between miR-29a-3p and both BASDAI and CRP, as well as associations between miR-222-3p, miR-625-3p and miR-885-5p and BASDAI. The data on miR-29a levels in AS are rather inconsistent. While miR-29a expression in peripheral blood mononuclear cells was lower in active AS patients than in controls and decreased after anti-TNF therapy [32], another showed otherwise [33]. TGF-β, an important stimulator of bone formation [34], inhibits miR-29a expression [35, 36]. In addition, miR-29a has been described as a negative regulator of Wnt signalling and production of extracellular matrix [35, 36]. We hypothesize that advanced-stage AS patients with extensive bone formation have higher levels of TGF-β, which ultimately results in miR-29a suppression and increased bone formation. MiR-222 has been predicted to exert inhibitory effects on genes associated with osteogenic differentiation [37], and low levels of miR-222 may result in increased MMP-13 expression in osteoarthritic cartilage [38]. As mentioned above, miR-625-3p was significantly decreased in nr-AxSpA and may be associated with early disease. There is no pathophysiologic explanation for this finding, nor is there an explanation for its association with BASDAI in advanced AS patients. Similarly, data regarding the potential role of miR-885-5p in AxSpA are lacking.

There are some limitations in our study. The selection of miRNAs for validation analysis may appear biased. The initial mirPath screen of all miRNAs of 1.5 mean fold difference obtained by TLDA provided us with a broad-spectrum unspecific data since such an online tool is based on validated/predicted targets genes coming from different fields (mostly cancer) that may be yet largely unknown in AxSpA. Due to practical reasons, it was not feasible to validate all miRNAs fulfilling the abovementioned cut-off. Therefore, a manual review was implemented to narrow down the list of miRNAs taken forward for further analysis and to enable drawing hypotheses. Next, two different normalization methods were used in our study. Array data were normalized to Ct average of all miRNAs as miRNAs of non-human origin are not included on the array platform, while the normalization to the average of 3 spike-in controls was performed in single-assay analysis. At present, there is no consensus on normalization of cell-free miRNAs. As the normalization to endogenous cell-free miRNAs may be conditioned by their altered expression due to (patho)physiological condition of each individual, the use of spike-in controls of non-human origin appears an acceptable alternative. Moreover, this approach reflects potential errors during the workflow, although these were minimized. We appreciate that the differential expression pattern based on TLDA analysis was not always reflected in single assay analysis. More studies involving larger patient cohorts are needed to confirm these data, as only a small proportion of the circulating mirnome has been analysed, and the functions of many miRNAs remain unknown. Moreover, due to nature of circulating miRNAs direct functional experiments are not feasible and the involvement of miRNAs in the pathogenesis of AS was hypothesized based on published data.

## Conclusions

We have shown for the first time the differential expression levels of several circulating miRNAs in radiographic AxSpA patients compared to non-radiographic disease patients and HC. Moreover, the levels of these miRNAs appear to reflect progressive spinal involvement. Interestingly, while some of these miRNAs play roles in bone formation and associated signalling pathways, others play roles in inflammation. It can be hypothesized that differential systemic miRNA expression levels reflect their dysregulation at sites of spinal inflammation or bone formation where they contribute to the development of pathophysiological features characteristic of AS. However, only miR625-3p, whose role in AS is unknown, exhibited significantly different expression levels in nr-AxSpA patients compared to HC and, interestingly, correlated with disease activity in AS. Our data support the role of circulating miRNAs in the pathogenesis of AxSpA and their potential as biomarkers of disease progression. Additional studies involving larger patient cohorts are needed to confirm these data, as only a small proportion of the circulating mirnome has been analysed, and the functions of many miRNAs remain unknown.

## Supporting information

S1 Fig The expression of miRNAs in healthy controls (HC) and patients with non-radiographic axial spondyloarthritis (nr-AxSpA), sacroiliitis and ankylosing spondylitis with spinal involvement (AS II-V) using TaqMan Low Density Array and their shared expression among groups. (TIF)

S1 Table Original Taq Man Low Density Array (TLDA) data obtained in 5 healthy controls (HC), and 5 patients with non-radiographic axial spondyloarthritis (nr-AxSpA), 5 patients with sacroiliitis and 5 patients with ankylosing spondylitis with spinal involvement (AS II-V). The statistical analysis was performed as described in Methods paragraph.
(XLSX)

S2 Table Functions of the 21 miRNAs selected for further analysis based on a literature search as explained in Methods paragraph. (DOCX)

S3 Table Data obtained from single assay analysis of 21 miRNAs. The data are provided as mean±SD calculated from data obtained from 29 healthy controls (HC), 20 patients with non-radiographic axial spondyloarthritis (nr-AxSpA), 24 patients with sacroiliitis and 24 patients with ankylosing spondylitis with spinal involvement (AS II-V). The statistical analysis was performed as described in Methods paragraph. Abbreviations: HC, healthy controls; nr-AxSpA, non-radiographic axial spondyloarthritis; AS, ankylosing spondylitis.
(DOCX)

S4 Table Extended summary of expression and function of selected miRNAs as markers of disease activity and hypothesized role in AxSpA. Abbreviations: HC, healthy controls; nr-AxSpA, non-radiographic axial spondyloarthritis; AS, ankylosing spondylitis; AS II-V, ankylosing spondylitis with spinal involvement; DMARDs, disease modifying antirheumaticdrugs; NSAID, non-steroidal anti-inflammatory drugs; T, T test; -, not significant. Statistical significance was calculated using ANOVA unless stated otherwise.
(DOCX)
