# Supplementary figures and images for: Association between circulating miRNAs and spinal involvement in patients with axial spondyloarthritis
Source: PLoS One. 2017 Sep 22;12(9):e0185323. doi: 10.1371/journal.pone.0185323 (PMC5609864; doi:10.1371/journal.pone.0185323)

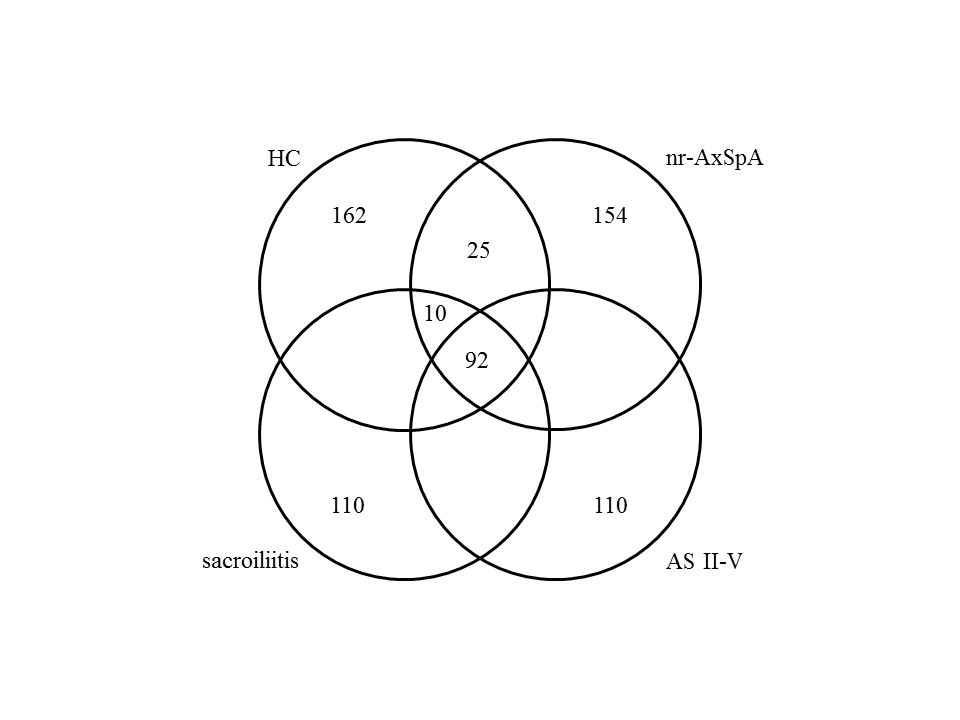

Supplement: S1 Fig — (TIF) [file pone.0185323.s001.tif]
