# Supplementary material for: Association between circulating miRNAs and spinal involvement in patients with axial spondyloarthritis
Source: PLoS One. 2017 Sep 22;12(9):e0185323. doi: 10.1371/journal.pone.0185323 (PMC5609864; doi:10.1371/journal.pone.0185323)
Supplement: S2 Table — (DOCX) [file pone.0185323.s003.docx]

**S2 Table. Functions of the 21 miRNAs selected for further analysis based on a literature search as explained in Methods paragraph.**

| **MiRNA** | **Brief description of function** |
| --- | --- |
| miR-19a-3p | negative regulator of Wnt pathway ^(^[^15^](#_ENREF_15)^)^ |
| miR-24-3p | osteogenic regulator^(^[^16^](#_ENREF_16)^)^ |
| miR-27a-3p | inhibition by osteoblastogenic factor Runx2 ^(^[^17^](#_ENREF_17)^)^, promoter of osteoblast differentiation ^(^[^18^](#_ENREF_18)^)^ |
| miR-29a-3p | inhibitor of Wnt pathway ^(^[^35^](#_ENREF_35)^)^, inhibition by osteogenic factor TGF-β ^(^[^36^](#_ENREF_36)^)^, biomarker of disease activity in AS ^(^[^32^](#_ENREF_32)^)^ |
| miR-99b-5p | promoter of osteoclast differentiation ^(^[^30^](#_ENREF_30)^)^ |
| miR-106a-5p | inhibitor of osteogenesis ^(^[^19^](#_ENREF_19)^)^, inhibitor of IL-8 ^(^[^20^](#_ENREF_20)^)^ |
| miR-133a-3p | proliferation and differentiation of myoblasts ^(^[^39^](#_ENREF_39)^)^ |
| miR-140-3p | inhibitor of cartilage degradation ^(^[^26^](#_ENREF_26)^)^,inhibitor of autoimmune arthritis ^(^[^27^](#_ENREF_27)^)^ |
| miR-145-5p | inhibitor of osteoblast differentiation ^(^[^31^](#_ENREF_31)^)^ |
| miR-146a-5p | inhibitor of osteoclastogenesis ^(^[^28^](#_ENREF_28)^)^, negative regulator of inflammation ^(^[^40^](#_ENREF_40)^)^, polymorphisms are a potential pathogenic factor for AS ^(^[^29^](#_ENREF_29)^)^ |
| miR-146b-5p | promoter of cell migration and invasion ^(^[^41^](#_ENREF_41)^)^ |
| miR-151a-3p | promoter of cell migration ^(^[^42^](#_ENREF_42)^)^ |
| miR-181a-5p | biomarker of inflammatory response, promoter osteoblast differentiation ^(^[^43^](#_ENREF_43)^)^ |
| miR-221-3p | overexpression in T cells of AS patients - immunopathogenesis of AS? ^(^[^44^](#_ENREF_44)^)^ |
| miR-222-3p | inhibitor of osteoblast differentiation ^(^[^37^](#_ENREF_37)^)^, regulation MMP-13 ^(^[^38^](#_ENREF_38)^)^ |
| miR-223-3p | inhibitor of osteoblast differentiation^(^[^22^](#_ENREF_22)^)^, promoter of osteoclastogenesis ^(^[^23^](#_ENREF_23)^)^ |
| miR-374a-5p | activator of Wnt pathway ^(^[^25^](#_ENREF_25)^)^ |
| miR-375 | inhibitor of osteogenesis ^(^[^45^](#_ENREF_45)^)^ |
| miR-409-3p | inhibitor of proliferation, vascularization and metastasis ^(^[^46^](#_ENREF_46)^)^ |
| miR-625-3p | diagnostic biomarker for mesothelioma ^(^[^47^](#_ENREF_47)^)^, marker of treatment response ^(^[^48^](#_ENREF_48)^)^ |
| miR-885-5p | predictor of knee or hip osteoarthritis ^(^[^49^](#_ENREF_49)^)^ |

39. Deng Z, Chen JF, Wang DZ. Transgenic overexpression of miR-133a in skeletal muscle. BMC musculoskeletal disorders. 2011;12:115.

40. Zhou Q, Haupt S, Kreuzer JT, Hammitzsch A, Proft F, Neumann C, et al. Decreased expression of miR-146a and miR-155 contributes to an abnormal Treg phenotype in patients with rheumatoid arthritis. Annals of the rheumatic diseases. 2015;74(6):1265-74.

41. Chou CK, Yang KD, Chou FF, Huang CC, Lan YW, Lee YF, et al. Prognostic implications of miR-146b expression and its functional role in papillary thyroid carcinoma. The Journal of clinical endocrinology and metabolism. 2013;98(2):E196-205.

42. Ding J, Huang S, Wu S, Zhao Y, Liang L, Yan M, et al. Gain of miR-151 on chromosome 8q24.3 facilitates tumour cell migration and spreading through downregulating RhoGDIA. Nat Cell Biol. 2010;12(4):390-9.

43. Bhushan R, Grunhagen J, Becker J, Robinson PN, Ott CE, Knaus P. miR-181a promotes osteoblastic differentiation through repression of TGF-beta signaling molecules. The international journal of biochemistry & cell biology. 2013;45(3):696-705.

44. Lai NS, Yu HC, Chen HC, Yu CL, Huang HB, Lu MC. Aberrant expression of microRNAs in T cells from patients with ankylosing spondylitis contributes to the immunopathogenesis. Clinical and experimental immunology. 2013;173(1):47-57.

45. Du F, Wu H, Zhou Z, Liu YU. microRNA-375 inhibits osteogenic differentiation by targeting runt-related transcription factor 2. Experimental and therapeutic medicine. 2015;10(1):207-12.

46. Weng C, Dong H, Chen G, Zhai Y, Bai R, Hu H, et al. miR-409-3p inhibits HT1080 cell proliferation, vascularization and metastasis by targeting angiogenin. Cancer letters. 2012;323(2):171-9.

47. Kirschner MB, Cheng YY, Badrian B, Kao SC, Creaney J, Edelman JJ, et al. Increased circulating miR-625-3p: a potential biomarker for patients with malignant pleural mesothelioma. Journal of thoracic oncology : official publication of the International Association for the Study of Lung Cancer. 2012;7(7):1184-91.

48. Rasmussen MH, Jensen NF, Tarpgaard LS, Qvortrup C, Romer MU, Stenvang J, et al. High expression of microRNA-625-3p is associated with poor response to first-line oxaliplatin based treatment of metastatic colorectal cancer. Molecular oncology. 2013;7(3):637-46.

49. Beyer C, Zampetaki A, Lin NY, Kleyer A, Perricone C, Iagnocco A, et al. Signature of circulating microRNAs in osteoarthritis. Annals of the rheumatic diseases. 2015;74(3):e18.
