# Supplementary material for: Association between circulating miRNAs and spinal involvement in patients with axial spondyloarthritis
Source: PLoS One. 2017 Sep 22;12(9):e0185323. doi: 10.1371/journal.pone.0185323 (PMC5609864; doi:10.1371/journal.pone.0185323)
Supplement: S3 Table — The data are provided as mean±SD calculated from data obtained from 29 healthy controls (HC), 20 patients with non-radiographic axial spondyloarthritis (nr-AxSpA), 24 patients with sacroiliitis and 24 patients with ankylosing spondylitis with spinal involvement (AS II-V). The statistical analysis was performed as described in Methods paragraph. Abbreviations: HC, healthy controls; nr-AxSpA, non-radiographic axial spondyloarthritis; AS, ankylosing spondylitis. (DOCX) [file pone.0185323.s004.docx]

**S3 Table. Data obtained from single assay analysis of 21 miRNAs.** The data are provided as mean±SD calculated from data obtained from 29 healthy controls (HC), 20 patients with non-radiographic axial spondyloarthritis (nr-AxSpA), 24 patients with sacroiliitis and 24 patients with ankylosing spondylitis with spinal involvement (AS stage II-V). The statistical analysis was performed as described in Methods paragraph.

| **miRNA** | **Relative expression dCt (mean±SD)** | | | | | |
| --- | --- | --- | --- | --- | --- | --- |
|  | **HC** | **nr-AxSpA** | **AS** | **sacroiliitis** | **AS II-V** | **Bamboo** |
| **miR-19a-3p** | -8.39 ± 2.19 | -8.13 ± 1.55 | -9.93 ± 1.35 | -9.60 ± 0.96 | -9.37 ± 0.92 | -12.22 ± 0.78 |
| **miR-24-3p** | -5.75 ± 2.39 | -5.63 ± 2.01 | -7.60 ± 1.18 | -7.18 ± 0-68 | -7.31 ± 1.05 | -9.56 ± 0.65 |
| **miR-27a-3p** | -7.29 ± 2.17 | -7.36 ± 1.93 | -9.12 ± 0.88 | -8.95 ± 0.63 | -8.83 ± 0.90 | -10.27 ± 0.65 |
| **miR-29a-3p** | -8.07 ± 1.50 | -8.42 ± 1.35 | -9.46 ± 0.71 | -9.39 ± 0.53 | -9.22 ± 0.62 | -10.20 ± 0.97 |
| **miR-99b-5p** | -10.27± 2.37 | -10.73 ± 1.75 | -12.24 ± 1.14 | -11.63 ± 0.57 | -12.36 ± 1.15 | -13.89 ± 0.75 |
| **miR-106a-5p** | -5.29 ± 2.00 | -4.90 ± 1.53 | -6.68 ± 0.99 | -6.32 ± 0.81 | -6.49 ± 0.71 | -8.21 ± 0.47 |
| **miR-133a-3p** | -11.87 ± 2.07 | -12.09 ± 2.55 | -13.34 ± 1.11 | -13.32 ± 0.99 | -13.09 ± 1.04 | -13.94 ± 1.57 |
| **miR-140-3p** | -11.97 ± 1.64 | -11.56 ± 1.05 | 13.03 ± 0.98 | -12.75 ± 0.76 | -12.76 ± 0.84 | -14.51 ± 0.53 |
| **miR-145-5p** | -9.39 ± 2.42 | -9.32 ± 2.17 | -10.83 ± 1.30 | -10.37 ± 0.09 | -10.85 ± 1.48 | -12.24 ± 1.14 |
| **miR-146a-5p** | -6.18 ± 2.33 | -6.06 ± 2.05 | -7.74 ± 0.84 | -7.52 ± 0.83 | -7.80 ± 0.88 | -8.30 ± 0.54 |
| **miR-146b-5p** | 8.27 ± 2.09 | -8.15 ± 1.54 | -9.74 ± 0.76 | -9.52 ± 0.59 | -9.54 ± 0.66 | -10.87 ± 0.31 |
| **miR-151a-3p** | -7.96 ± 2.39 | -7.75 ± 2.16 | -9.34 ± 0.93 | -9.23 ± 0.91 | -9.52 ± 0.97 | -9.32 ± 1.04 |
| **miR-181a-5p** | -10.60 ± 2.04 | -10.83 ± 1.78 | -11.53 ± 0.93 | -11.35 ± 0.81 | -11.61 ± 1.14 | -11.92 ± 0.80 |
| **miR-221-3p** | -6.07 ± 2.57 | -5.88± 2.30 | -7.71 ± 0.96 | -7.59 ± 0.86 | -7.80 ± 1.16 | -7.88 ± 0.90 |
| **miR-222-3p** | -1.40 ± 0.65 | -2.03 ± 1.02 | -3.03 ± 1.40 | -2.64 ± 1.18 | -2.80 ± 1.16 | -4.76 ± 1.42 |
| **miR-223-3p** | -2.94 ± 2.55 | -2.89 ± 2.24 | -4.71 ± 1.30 | -4.37 ± 0.87 | -4.37 ± 1.32 | -6.51 ± 0.95 |
| **miR-374a-5p** | -10.92 ± 2.98 | -11.52 ± 2.51 | -13.41 ± 1.17 | -13.02 ± 0.87 | -13.19 ± 0.87 | -15.10 ± 1.24 |
| **miR-375** | -11.26 ± 1.11 | -11.29 ± 1.42 | -12.22 ± 1.43 | -11.72 ± 1.34 | -12.16 ± 1.18 | -13,87 ± 1.09 |
| **miR-409-3p** | -8.19 ± 2.38 | -8.99 ± 1.94 | -10.11 ± 0.94 | -10.43 ± 0.760 | -9.98 ± 1.11 | -10.56 ± 1.09 |
| **miR-625-3p** | 1.85 ± 0.52 | 0.64 ± 1.41 | 0.06 ± 1.47 | 0.64 ± 1.19 | 0.11 ± 1.14 | -1.85 ± 1.43 |
| **miR-885-5p** | -10.88 ± 1.68 | -10.66 ± 1.65 | -13.66 ± 0.55 | -10.27 ± 1.83 | -11.20 ± 1.00 | -13.66 ± 0.55 |

Abbreviations: HC, healthy controls; nr-AxSpA, non-radiographic axial spondyloarthritis; AS, ankylosing spondylitis.
