# Supplementary material for: Association between circulating miRNAs and spinal involvement in patients with axial spondyloarthritis
Source: PLoS One. 2017 Sep 22;12(9):e0185323. doi: 10.1371/journal.pone.0185323 (PMC5609864; doi:10.1371/journal.pone.0185323)
Supplement: S4 Table — Abbreviations: HC, healthy controls; nr-AxSpA, non-radiographic axial spondyloarthritis; AS, ankylosing spondylitis; AS II-V, ankylosing spondylitis with spinal involvement; DMARDs, disease modifying antirheumaticdrugs; NSAID, non-steroidal anti-inflammatory drugs; T, T test; -, not significant. Statistical significance was calculated using ANOVA unless stated otherwise. (DOCX) [file pone.0185323.s005.docx]

**S4 Table. Selected miRNAs as markers of disease activity and hypothesized role in AxSpA**

| **miRNA** | **Diagnosis** | | | | **Structural damage** | | | | **Disease activity** | | | | **Treatment response** | | **Hypothesized role**  **in AxSpA** |
| --- | --- | --- | --- | --- | --- | --- | --- | --- | --- | --- | --- | --- | --- | --- | --- |
|  | **HC**  **vs.**  **AxSpA^T^** | **HC**  **vs.**  **nr-AxSpA** | **HC**  **vs.**  **AS** | **nr-AxSpA vs.**  **AS** | **nr-AxSpA vs.**  **sacroiliitis** | **nr-AxSpA vs.**  **AS II-V** | **sacroiliitis**  **vs.**  **AS II-V** | **AS**  **vs.**  **bamboo^T^** | **nr-AxSpA** | **AS** | **sacroiliitis** | **AS II-V** | **NSAID**  **vs.**  **antiTNF** | **DMARDs.**  **vs.**  **antiTNF** |  |
| **miR-19a-3p** | 0.045 | - | 0.006 | 0.013 | 0.016 | ≤0.001 | - | ≤0.001 | - | - | - | - | - | 0.003 | bone formation |
| **miR-24-3p** | 0.022 | - | 0.002 | 0.010 | 0.013 | ≤0.001 | - | ≤0.001 | - | - | - | - | ≤0.001 | ≤0.001 | bone formation |
| **miR-27a-3p** | 0.010 | - | ≤0.001 | 0.010 | 0.003 | ≤0.001 | - | 0.002 | - | - | - | - | ≤0.001 | 0.010 | bone formation |
| **miR-29a-3p** | 0.003 | - | ≤0.001 | 0.039 | 0.018 | 0.006 | - | - | - | - | - | BASDAI, CRP | 0.040 | 0.003 | bone formation, |
| **miR-99b-5p** | 0.006 | - | ≤0.001 | 0.049 | - | ≤0.001 | 0.011 | ≤0.001 | CRP | BASDAI | - | - | ≤0.001 | 0.003 | bone formation |
| **miR-106a-5p** | - | - | 0.005 | 0.004 | 0.003 | ≤0.001 | - | ≤0.001 | - | - | - | - | ≤0.001 | ≤0.001 | bone formation |
| **miR-133a-3p** | 0.016 | - | 0.016 | - | - | - | - | - | - | BASDAI | - | CRP | - | 0.043 | differentiation ? |
| **miR-140-3p** | - | - | 0.011 | 0.005 | 0.005 | ≤0.001 | - | ≤0.001 | CRP | - | - | - | 0.002 | 0.005 | inflammation |
| **miR-145-5p** | - | - | 0.026 | - | - | 0.003 | - | 0.013 | CRP | - | - | - | 0.001 | 0.023 | bone formation |
| **miR-146a-5p** | 0.044 | - | 0.006 | 0.021 | 0.009 | ≤0.001 | - | 0.031 | - | CRP | - | - | 0.013 | 0.010 | inflammation |
| **miR-146b-5p** | 0.035 | - | 0.002 | 0.010 | 0.002 | ≤0.001 | - | ≤0.001 | - | - | - | - | 0.005 | ≤0.001 | migration, invasion |
| **miR-151a-3p** | - | - | 0.022 | 0.040 | 0.014 | 0.004 | - | - | - | CRP | - | CRP | - | - | migration |
| **miR-181a-5p** | - | - | - | - | - | - | - | - | - | CRP | - | - | - | - | ? |
| **miR-221-3p** | - | - | 0.010 | 0.025 | 0.007 | 0.002 | - | - | - | CRP | - | - | - | 0.012 | immunopathogenesis |
| **miR-222-3p** | ≤0.001 | - | ≤0.001 | 0.049 | - | 0.012 | - | 0.015 | - | - | - | BASDAI | - | - | bone formation |
| **miR-223-3p** | 0.036 | - | 0.007 | 0.034 | 0.037 | 0.002 | - | 0.001 | - | - | - | - | 0.004 | 0.004 | bone formation |
| **miR-374a-5p** | 0.006 | - | ≤0.001 | 0.050 | 0.04 | 0.001 | - | 0.009 | CRP | - | - | - | 0.018 | 0.029 | bone formation |
| **miR-375** | 0.045 | - | - | - | - | 0.020 | - | 0.005 | - | - | - | BASDAI | - | - | bone formation |
| **miR-409-3p** | 0.005 | - | ≤0.001 | - | - | 0.046 | - | - | - | - | - | CRP | - | - | proliferation, invasion |
| **miR-625-3p** | ≤0.001 | 0.024 | ≤0.001 | - | - | - | 0.033 | 0.009 | - | BASDAI | - | BASDAI | - | - | ? |
| **miR-885-5p** | - | - | - | - | - | - | 0.009 | ≤0.001 | - | BASDAI | - | BASDAI | - | - | ? |

Abbreviations: HC, healthy controls; nr-AxSpA, non-radiographic axial spondyloarthritis; AS, ankylosing spondylitis; AS II-V, ankylosing spondylitis with spinal involvement; DMARDs, disease modifying antirheumaticdrugs; NSAID, non-steroidal anti-inflammatory drugs; T, T test; -, not significant; Statistical significance was calculated using ANOVA unless stated otherwise.
